# Prevalence of abnormal Alzheimer’s disease biomarkers in patients with subjective cognitive decline: cross-sectional comparison of three European memory clinic samples

**DOI:** 10.1186/s13195-018-0463-y

**Published:** 2019-01-17

**Authors:** Steffen Wolfsgruber, José Luis Molinuevo, Michael Wagner, Charlotte E. Teunissen, Lorena Rami, Nina Coll-Padrós, Femke H. Bouwman, Rosalinde E. R. Slot, Linda M. P. Wesselman, Oliver Peters, Katja Luther, Katharina Buerger, Josef Priller, Christoph Laske, Stefan Teipel, Annika Spottke, Michael T. Heneka, Emrah Düzel, Alexander Drzezga, Jens Wiltfang, Sietske A. M. Sikkes, Wiesje M. van der Flier, Frank Jessen, on behalf of the Euro-SCD working group

**Affiliations:** 1 Department of Neurodegeneration and Geriatric Psychiatry, University Hospital Bonn, Bonn, Germany; 2 German Center for Neurodegenerative Diseases, Sigmund-Freud-Straße 27, 53127 Bonn, Germany; 3 Alzheimer’s Disease and Other Cognitive Disorders Unit, IDIBAPS, Hospital Clinic, Barcelona, Spain; 4 BarcelonaBeta Brain Research Center, Fundació Pasqual Maragall, Pompeu Fabra University, Barcelona, Spain; 5 Neurochemistry Lab and Biobank, Department of Clinical Chemistry, Amsterdam Neuroscience, Amsterdam University Medical Centers, Amsterdam, The Netherlands; 6 Alzheimer Center and Department of Neurology, Neuroscience Campus Amsterdam, Amsterdam University Medical Centers, Amsterdam, The Netherlands; 7 Department of Psychiatry and Psychotherapy, Charité, Campus Benjamin Franklin, Berlin, Germany; 8 Institute for Stroke and Dementia Research, Klinikum der Universität München, Ludwig-Maximilians-Universität, Munich, Germany; 9 German Center for Neurodegenerative Diseases, Munich, Germany; 10 Department of Neuropsychiatry, Charité-Universitaetsmedizin Berlin, Berlin, Germany; 11 Department of Psychiatry and Psychotherapy, University of Tübingen, Tübingen, Germany; 12 German Center for Neurodegenerative Diseases, Tübingen, Germany; 13 German Center for Neurodegenerative Diseases, Rostock, Germany; 14 German Center for Neurodegenerative Diseases, Magdeburg, Germany; 15 Department of Nuclear Medicine, Medical Faculty, University of Cologne, Cologne, Germany; 16 German Center for Neurodegenerative Diseases (DZNE), Goettingen, Germany; 17 Department of Psychiatry and Psychotherapy, University Medical Center Goettingen, Goettingen, Germany; 18 Department of Epidemiology and Biostatistics, Amsterdam University Medical Centers, Amsterdam, The Netherlands; 19 Massachusetts General Hospital, Department of Neurology / Harvard Medical School, Boston, USA; 20 Department of Psychiatry, Medical Faculty, University of Cologne, Cologne, Germany; 21 iBiMED, Medical Sciences Department, University of Aveiro, Aveiro, Portugal

**Keywords:** Subjective cognitive decline, Preclinical Alzheimer’s disease, CSF biomarkers

## Abstract

**Introduction:**

Subjective cognitive decline (SCD) in cognitively unimpaired older individuals has been recognized as an early clinical at-risk state for Alzheimer’s disease (AD) dementia and as a target population for future dementia prevention trials. Currently, however, SCD is heterogeneously defined across studies, potentially leading to variations in the prevalence of AD pathology. Here, we compared the prevalence and identified common determinants of abnormal AD biomarkers in SCD across three European memory clinics participating in the European initiative on harmonization of SCD in preclinical AD (Euro-SCD).

**Methods:**

We included three memory clinic SCD samples with available cerebrospinal fluid (CSF) biomaterial (IDIBAPS, Barcelona, Spain, *n* = 44; Amsterdam Dementia Cohort (ADC), The Netherlands, *n* = 50; DELCODE multicenter study, Germany, *n* = 42). CSF biomarkers (amyloid beta (Aβ)42, tau, and phosphorylated tau (ptau181)) were centrally analyzed in Amsterdam using prespecified cutoffs to define prevalence of pathological biomarker concentrations. We used logistic regression analysis in the combined sample across the three centers to investigate center effects with regard to likelihood of biomarker abnormality while taking potential common predictors (e.g., age, sex, apolipoprotein E (APOE) status, subtle cognitive deficits, depressive symptoms) into account.

**Results:**

The prevalence of abnormal Aβ42, but not tau or ptau181, levels was different across centers (64% DELCODE, 57% IDIBAPS, 22% ADC; *p* < 0.001). Logistic regression analysis revealed that the likelihood of abnormal Aβ42 (and also abnormal tau or ptau181) levels was predicted by age and APOE status. For Aβ42 abnormality, we additionally observed a center effect, indicating between-center heterogeneity not explained by age, APOE, or the other included covariates.

**Conclusions:**

While heterogeneous frequency of abnormal Aβ42 was partly explained by between-sample differences in age range and APOE status, the additional observation of center effects indicates between-center heterogeneity that may be attributed to different recruitment procedures. These findings highlight the need for the development of harmonized recruitment protocols for SCD case definition in multinational studies to achieve similar enrichment rates of preclinical AD.

## Background

It is widely acknowledged that future prevention and intervention approaches for Alzheimer’s disease (AD) will be most effective when applied to individuals in a disease stage prior to mild cognitive impairment (MCI) or prodromal AD [1, 2]. As in the latest research framework guidelines proposed for observational and intervention studies [3], AD is defined in vivo by profiling of biomarkers (e.g., those obtained from cerebrospinal fluid (CSF)) grouped into those of amyloid beta deposition (A), pathological tau (T), and those of neurodegeneration (N) in the “AT(N)” system. Preclinical AD is present if patients are cognitively unimpaired and have a biomarker profile of both abnormality in amyloid beta (A^+^) and pathological tau markers (T^+^). Importantly, in this new framework, subjective cognitive decline (SCD) is considered indicative of a stage of transitional cognitive decline, that is between a fully asymptomatic stage and a cognitively impaired (MCI) stage of the disease.

Thus, SCD in cognitively unimpaired older individuals is recognized as a pre-MCI at-risk state of AD dementia and a target condition for future AD dementia prevention trials. A first set of consensus criteria and research guidelines for operationalization of SCD have been published, but comparability of SCD samples across different research sites is still poor [4, 5]. Conditions other than AD may cause symptoms of SCD which further promotes heterogeneity. This could be countered by harmonized recruitment protocols including aspects of SCD that enhance the likelihood of underlying AD [4, 5]. However, there is currently a lack of such protocols for SCD case definition and assessment in the context of preclinical AD. The design of such a protocol represents a crucial next step before applying SCD in largescale AD prevention trials. To address this issue, the European initiative on harmonization of SCD in preclinical AD (Euro-SCD) aims to develop a harmonized multicenter, multinational case-definition protocol of SCD which should yield comparable rates of preclinical AD (i.e., similar enrichment for AD risk) across memory clinic cohorts. A first step of EURO-SCD, presented in this study, is to retrospectively analyze data from the memory clinic cohorts of the three participating study partners, each recruited with their own SCD recruitment protocols. Here, our aim was to evaluate the extent of heterogeneity in biomarker abnormality across the three European SCD samples and identifying potential sources for this.

## Methods

### Standard protocol approvals, registrations, and patient consent

The study protocol was approved by the Institutional Review Boards of all participating study centers of the Euro-SCD project. All patients provided written informed consent.

### Participants

We analyzed data from three ongoing memory clinic cohorts that recruit SCD participants and collaborate within EURO-SCD: The Amsterdam Dementia Cohort (ADC) [6, 7], the cohort of l’Institut d’Investigacions Biomèdiques August Pi i Sunyer Hospital Clinic Barcelona (IDIBAPS) [8], and the German Center for Neurodegenerative Diseases multicenter Longitudinal Cognitive Impairment and Dementia Study (DELCODE) [9]. Each cohort was asked to contribute a target number of 50 or close to 50 SCD patients with available CSF samples. All CSF samples were then centrally analyzed (in Amsterdam) with regard to AD biomarkers as detailed below. Each cohort is briefly described below, together with its respective SCD recruitment protocol and case definition.

### Recruitment procedures and case definition of SCD in each sample

#### DELCODE cohort

DELCODE is an observational longitudinal memory clinic-based multicenter study carried out by 10 university memory clinics, based within the German Center for Neurodegenerative Diseases (DZNE) research infrastructure. Baseline recruitment started in 2015 and is ongoing. A complete description of DELCODE has been published previously [9]. All SCD patients are referrals, including self-referrals, and all were assessed clinically at the respective memory clinics before entering the study. Assessments included medical history, psychiatric and neurological examination, neuropsychological testing, blood laboratory work-up, and routine magnetic resonance imaging (MRI). The German version of the consortium to establish a registry of Alzheimer’s disease (CERAD) neuropsychological test battery [10], which includes the Trail-Making Test (TMT) A and B [11], was applied at all memory centers. SCD was defined by the presence of subjectively reported decline in cognitive functioning, experienced as worrisome, and a test performance of better than −1.5 standard deviations (SDs) below age-, education-, and gender-adjusted normal performance [12] on all subtests of the CERAD neuropsychological battery, in line with current SCD research criteria [5]. Additional inclusion criteria were age ≥ 60 years, fluent German language skills, capacity to provide informed consent, and the presence of a study partner. Main exclusion criteria were conditions which clearly interfere with participation in the study or the study procedures, for example significant sensory impairment, current major depressive episode or other major psychiatric disorders either at baseline or in the past, and chronic use of psychoactive compounds with sedative or anticholinergic effects (see [9] for a full list of inclusion/exclusion criteria).

#### Amsterdam Dementia Cohort (ADC)

The ADC is a cohort consisting of consecutive patients visiting the Alzheimer Center of the VU University Medical Center (VUmc) in Amsterdam, the Netherlands. It has been described in detail previously [6, 7]. All SCD patients underwent a standardized dementia screening, including physical and neurologic examination as well as laboratory tests and brain MRI. Cognitive assessment included the Mini-Mental State Examination (MMSE) and an extensive neuropsychological test battery. Diagnoses were made in a multidisciplinary case conference. Patients were defined as SCD when they presented with cognitive complaints, but cognitive and laboratory investigations were normal and criteria for MCI, dementia, or any other neurologic or psychiatric disorders known to cause cognitive complaints were not met. Petersen’s criteria were used for MCI [13], where the presence of MCI-like objective cognitive impairment was determined by clinical judgment of the complete neuropsychological information rather than applying a specific algorithm or impairment cutoff.

#### IDIBAPS Barcelona cohort

The biomarker cohort of the IDIBAPS Hospital Clinic Barcelona recruited patients with SCD, MCI, and AD dementia, as well as cognitively unimpaired participants without cognitive complaints. It has been described in detail previously [8].

Subjects with any neurological diagnosis, serious, or unstable medical condition, or with a diagnosis of major psychiatric disorder including schizophrenia and major depressive, severe somatic disease, or substance abuse were excluded in all the groups. The clinical SCD group comprises subjects who consulted the IDIBAPS Hospital Clinic memory clinic for experience of subjective cognitive decline. They presented normal scores on two screening tests, namely MMSE and the Memory Alteration Test (M@T) [14], and on all subtests of a neuropsychological battery tapping cognitive domains of memory, language, praxis, visuo-perceptive and/or visuospatial ability, and executive functions. Similar to DELCODE, test performance of better than −1.5 SD below the mean of healthy controls, matched for age and education, in all subtests of the applied neuropsychological battery was required for a study diagnosis of SCD.

### CSF measures

#### CSF sampling and analytic procedures

CSF was obtained via lumbar puncture using a 25-gauge needle and collected in 10-ml polypropylene tubes (Sarstedt, Nümbrecht, Germany) in agreement with international consensus protocols [15]. Within 2 h, CSF samples were centrifuged at 4 °C for 10 min at 1800 g. CSF supernatant was transferred to 0.5-ml polypropylene tubes and stored at −20 °C until further analysis (within 2 months) for the Amsterdam samples. Samples collected in the IDIBAPS and DELCODE cohort were stored at −80 °C until transfer to Amsterdam for central analysis. Commercially available enzyme-linked immunosorbent assays (ELISAs) (Innotest β-amyloid(1–42), InnotestTAU-Ag, and InnotestPhosphotau(181P); Fujirebio, Ghent, Belgium) were applied to measure baseline amyloid beta (Aβ)42, total tau (t-tau), and tau phosphorylated as position 181 (ptau181) as previously described elsewhere [16, 17]. Clinical diagnosis was unknown to the team performing the CSF analyses.

#### Definition of AD biomarker abnormality

For definition of AD biomarker abnormality, we applied previously published cutoff values: abnormal CSF-Aβ42 was defined as values < 813 pg/ml [17]; abnormal CSF t-tau was defined as values > 375 pg/ml; and CSF-ptau181 was defined as values > 53 pg/ml [18]. Besides abnormality in individual markers, we also report results for a CSF-based operationalization of preclinical AD according to the most recent National Institute on Aging and the Alzheimer’s Association (NIA-AA) criteria [3], that is defined by the presence of both abnormal CSF-Aβ42 and CSF-ptau181.

### Clinical and neuropsychological assessment

Clinical and neuropsychological assessment was carried out in each center following center-specific standardized diagnostic procedures that have been described in other publications (ADC [6, 7], IDIBAPS [19], and DELCODE [9]). Here, we only report the assessments relevant to the present study. For the clinical and neuropsychological data to be used as predictors of CSF abnormality across samples, we applied several rescaling procedures as described below.

#### Assessment of neuropsychological test performance

All centers applied established neuropsychological tests mainly covering three cognitive domains. Verbal memory was assessed with the German Version of the CERAD wordlist [10] in DELCODE, the Dutch version of the Rey Verbal Learning Test (RVLT) [20] in ADC, and the Spanish version of the Free and Cued Selective Reminding Test (FCSRT) [21] in the IDIBAPS sample. Executive functions and speed were measured by the TMT-A and TMT-B [11] in all three samples. Language abilities were measured by tests of semantic verbal fluency (animals) in all three samples [22]. The DELCODE and IDIBAPS sample also applied the 15-item version of the Boston Naming Test (BNT) [22], while a second, verbal fluency measure (letters) was available in the ADC sample [22].

We used center-specific normative data to derive age-, sex-, and education-adjusted *z*-scores for each sample. For each participant we then aggregated the *z*-score information of the available tests in the three cognitive domains into a single, dichotomized variable with the categories “evidence of subtle cognitive decline” vs. “no evidence of subtle cognitive decline”. We derived this variable by adapting the method proposed by Edmonds and colleagues [23]: “evidence of subtle cognitive decline” was defined by performance of more than 1 SD below the normative mean (i.e., a *z*-score < −1) on at least two out of six preselected neuropsychological measures (two of each of the three different cognitive domains described above). For verbal memory we used the word list delayed recall and recognition scores from the CERAD in DELCODE and from the RVLT in the ADC sample, respectively. The best equivalent to this in the IDIBAPS sample was the FCSRT free and total recall score [24]. In the language abilities domain, we utilized the animal fluency and BNT score in IDIBAPS and DELCODE, and animal + letter fluency score in the ADC sample.

#### Depressive symptomatology and instrumental activities of daily living performance

Depressive symptomatology was measured with the 15-item version of the Geriatric Depression Scale (GDS) [25] in DELCODE and ADC where a cutoff > 5 indicates depressive symptomatology. In IDIBAPS, depressive symptoms were measured using the Hospital Anxiety and Depression Scale (HADS) [26], where a cutoff > 7 indicates depressive symptomatology.

Instrumental activities of daily living (IADL) were assessed with the Functional Activities Questionnaire (FAQ) [27] in IDIBAPS and DELCODE and with the Disability Assessment for Dementia (DAD) scale in the ADC sample [28]. Due to the limited range and variance in SCD patients on these two respective measures, we derived a dichotomized variable with the following categories: “no IADL deficits” (fully unimpaired) vs. “subtle IADL deficits” (mildly imperfect performance, that is a score ≥ 1 on the FAQ or a score < 100 on the DAD, respectively).

### Statistical analysis

Statistical analyses were conducted with SPSS version 22. As this is an exploratory rather than a confirmatory analysis, we report unadjusted *p* values.

Our main analysis focused on evaluating heterogeneity of biomarker abnormality across the three subsamples and identifying potential sources for this. In that regard, we conducted four separate stepwise logistic regression analyses, i.e., one analysis each for abnormal CSF-Aβ42, CSF-tau, CSF-ptau181, and the aforementioned NIA-AA preclinical AD definition (i.e., presence of abnormal CSF-Aβ42 together with abnormal CSF-ptau181; [3]) as the dependent variable, respectively.

We included the following covariates/predictors in a forward selection procedure (*p* value for inclusion ≤ 0.05) in step one: age, sex, years of education, evidence of subtle cognitive decline, and apolipoprotein E (APOE) genotype. For insignificant predictors we will report the chi-squared values of the score test with the corresponding *p* values. The score test, or Lagrange multiplier test, is used in forward selection procedures to test for improvement of model fit if variables are successively added to a prediction model. If there was any cross-center heterogeneity in biomarker abnormality not explained by the predictors of step one, then a categorical predictor of “study-center” should significantly improve model fit by increasing explained variance beyond that of the covariate model. Therefore, we entered study center as an additional predictor in a second step. This main analysis was based on a sample of *n* = 132 cases (four cases with missing APOE).

In a supplementary analysis, we repeated the aforementioned regression models with additional inclusion of depressive symptomatology and subtle IADL deficits as predictors in step one. This was based on a restricted sample with *n* = 92 cases (*n* = 44 cases not included due to missing data on IADL performance (*n* = 39), depressive symptoms (*n* = 10), and/or APOE (*n* = 4); some subjects had missing values on more than one of these variables).

Cases with missing data did not differ regarding age, education, MMSE score, sex or (for those with missing on depression or IADL only) APOE status, suggesting that the assumption of a missing (completely) at random data pattern was not violated, thus allowing for either multiple imputation of the missing depression and IADL scale values or a complete case analysis. For the sake of simplicity, we report on the latter, as a sensitivity analysis using multiple imputation did not change the results.

## Results

### Descriptive statistics

Descriptive statistics of the whole sample and each subsample regarding demographical, clinical, neuropsychological, and biomarker data are given in Table 1.

**Table 1:** Baseline characteristics of the whole study sample

Characteristic	Whole sample (*n* = 136)	DELCODE (*n* = 42)	IDIBAPS (*n* = 44)	ADC(*n* = 50)	Between center differences (ANOVA/χ^2^)	
					F/χ^2^_(df = 3)_	*p* value
Age, years	66.5 ± 7.0	71.2 ± 4.8	66.2 ± 7.2	62.9 ± 6.1	21.0	**< 0.001**
Education, years	12.5 ± 4.2	14.9 ± 3.4	10.3 ± 4.6	12.4 ± 3.2	15.6	**< 0.001**
Sex, female (%)	51.5	42.9	79.5	34.0	21.2	**< 0.001**
MMSE total score	28.4 ± 1.5	29.0 ± 1.0	28.0 ± 1.7	28.1 ± 1.6	6.8	**0.002**
FAQ score^a^	–	0.80 ± 1.10	1.05 ± 1.75	–	–	–
DAD score^b^	–	–	–	96.70 ± 5.70	–	–
Subtle IADL deficits^c^ (%)	45.4	46.3	47.6	42.9	0.148	0.929
Evidence of depressive symptoms^d^ (%)	8.80	0.0	17.9	10.9	7.6	**0.022**
Verbal Delayed Recall, *z*-score	−0.04 ± 1.11	0.33 ± 1.00	0.11 ± 0.98	−0.49 ± 1.17	7.5	**0.001**
Recognition/Cued Recall, *z*-score	0.27 ± 0.90	0.49 ± 0.57	0.35 ± 1.01	0.00 ± 0.96	3.8	**0.025**
Verbal Fluency Animals, *z*-score	0.00 ± 0.91	0.31 ± 0.96	0.06 ± 0.73	−0.31 ± 0.92	5.6	**0.005**
Verbal Fluency Letters^b^, *z*-score	–	–	–	0.36 ± 1.12	–	–
Boston Naming ^a^, *z*-score	–	0.63 ± 0.65	0.30 ± 0.83	–	–	–
TMT-A, *z*-score	−0.09 ± 1.04	0.30 ± 1.20	0.02 ± 0.97	−0.50 ± 0.81	7.7	**0.001**
TMT-B, *z*-score	−0.15 ± 1.00	0.36 ± 1.09	−0.40 ± 1.01	−0.39 ± 0.72	9.3	**< 0.001**
Evidence of subtle cognitive decline^e^ (%)	22.8	14.3	9.1	42.0	16.9	**0.001**
Apolipoprotein E ε4 carriers (%)	35.6	28.2	32.6	44.0	2.6	0.267
Aβ42, pg/ml	860.8 ± 298.1	740.0 ± 216.8	741.0 ± 236.2	1067.8 ± 295.9	7,8	**< 0.001**
Aβ42 < 813 pg/ml (%)	46.3	64.3	56.8	22.0	19.3	**< 0.001**
t-tau, pg/ml	352.5 ± 280.8	322.4 ± 193.4	378.5 ± 314.6	354.9 ± 312.6	0.428	0.653
t-tau > 470 pg/ml (%)	25.7	31.0	29.5	18.0	2.5	0.287
ptau181, pg/ml	55.5 ± 28.7	51.9 ± 22.4	59.2 ± 34.2	55.4 ± 28.4	0.684	0.506
ptau181 > 57 pg/ml (%)	40.4	35.7	45.5	40.0	0.853	0.653
Aβ42 and ptau181 abnormal (%)	16.2	21.4	20.5	8.0	3.9	0.141

Table shows sample description for the whole study sample and each center’s subsample of SCD participants

Values are shown as mean ± standard deviation unless otherwise indicated

Significant *p* values are indicated in bold typeface

*Z*-scores for neuropsychological variables represent age-, sex-, and education-adjusted normative values after applying center-specific norms in all study sites (see Methods section for further details)

*Aβ* amyloid beta, *ADC* Amsterdam Dementia Cohort, *ANOVA* analysis of variance, *DAD* Disability Assessment for Dementia, *DELCODE* German Center for Neurodegenerative Diseases (DZNE) multicenter Longitudinal Cognitive Impairment and Dementia Study, *FAQ* Functional Activities Questionnaire, *GDS* Geriatric Depression Scale, *HADS* Hospital Anxiety and Depression Scale, *IADL* instrumental activities of daily living, *IDIBAPS* l’Institut d’Investigacions Biomèdiques August Pi i Sunyer Hospital Clinic Barcelona, *MMSE* Mini-Mental State Examination, *ptau181* tau phosphorylated at position 181, *TMT* Trail-Making Test, *t-tau* total tau

^a^ Assessed only in DELCODE and IDIBAPS

^b^ Assessed only in ADC

^c^ Subtle IADL deficits defined as mildly imperfect performance, i.e., a score ≥ 1 on the FAQ, or a score < 100 on the DAD, respectively

^d^ Evidence of depressive symptoms defined as a depression score above the cutoff of the applied scale (DELCODE and ADC, GDS > 5; IDIBAPS, HADS > 7)

^e^ Evidence of subtle cognitive decline operationalized according to criteria proposed by Edmonds and colleagues (see [23] and Methods section): impaired score of >1 SD below normative mean (i.e., *z*-score < −1) on two measures in different cognitive domains

The mean age substantially varied between the centers, with the ADC participants (mean ± SD, 62.9 ± 6.1 years) being on average about 3.5 years younger than those of IDIBAPS (66.2 ± 7.2 years) and about 8 years younger than those of DELCODE (71.2 ± 4.8). We further observed differences between the samples regarding sex (with IDIBAPS containing a substantially higher number of women), education, frequency of subclinical depressive symptomatology, and neuropsychological variables. Frequency of positive APOE4 status (overall 35.6%) did not differ between the three samples. As expected, neuropsychological performance was in the range of cognitive normality, with average norm-adjusted *z*-scores between +0.5 and −0.5 SD, in all samples. However, there was still variation within this range as indicated by significant between-center differences in the number of participants meeting the neuropsychological criteria [23] used to define “evidence of subtle cognitive decline” (9.1% in IDIBAPS, 14.3% in DELCODE, and 42% in ADC, *p* = 0.001).

**Fig. 1:**
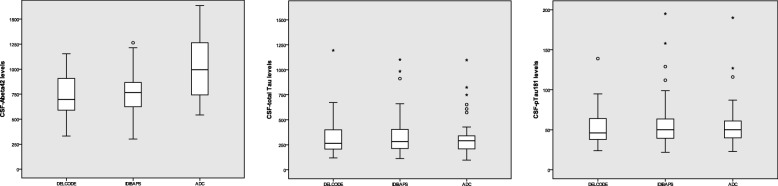
Boxplots for cerebrospinal fluid (CSF) levels in each sample of the three participating Euro-SCD centers. Values are presented in pg/ml. Points mark individual values lying outside 1.5 times the interquartile range (25–75% percentile). Asterisks mark extreme values lying outside three times the interquartile range. ^#^The DELCODE and IDIBAPS sample show significantly lower levels compared with the ADC sample (*p* < 0.001). ADC Amsterdam Dementia Cohort, DELCODE German Center for Neurodegenerative Diseases (DZNE) multicenter Longitudinal Cognitive Impairment and Dementia Study, IDIBAPS l’Institut d’Investigacions Biomèdiques August Pi i Sunyer Hospital Clinic Barcelona

Regarding the CSF biomarkers, distribution for each center in the form of boxplots is shown in Fig. 1. We observed (pooled) frequencies of abnormal CSF-Aβ42 in 46.3%, abnormal CSF-tau in 25.7%, and abnormal CSF-ptau181 in 40.4% of all SCD patients. CSF-defined preclinical AD (both abnormal CSF-Aβ42 and CSF-ptau181) was observed in 18.4%.

### Between-center heterogeneity in AD biomarker abnormality

On a descriptive level (i.e., not accounting for covariates) only the CSF-Aβ42 levels were significantly different across the centers, both in terms of continuous values and frequency of abnormal CSF-Aβ42 according to the applied cutoff (< 813 pg/ml; 64% DELCODE, 57% IDIBAPS, 22% ADC; *p* < 0.001, see Table 1 and Fig. 1).

Results of the logistic regression analyses for prediction of each abnormal CSF biomarker are presented in Table 2. Higher age and a positive APOE status (i.e., at least one E4 allele) were associated with a higher likelihood of CSF-Aβ42 abnormality. Sex (score test χ^2^_(df = 1)_ = 0.027, *p* = 0.871), years of education (score test χ^2^_(df = 1)_ = 0.334, *p* = 0.563), and evidence of subtle cognitive decline (score test χ^2^_(df = 1)_ = 0.044, *p* = 0.833) did not show a significant association.

**Table 2:** Results of logistic regression analysis for prediction of abnormal CSF biomarkers across all three centers

Model/predictors	OR	(95% CI)		*p* value	*R* ^2^	Δ*R*^2^	OR	(95% CI)		*p* value	*R* ^2^	Δ*R*^2^
Model variables	Abnormal CSF-Aβ42 (< 813 pg/ml)						Abnormal CSF-tau (> 375 pg/ml)					
Step 1: covariates												
Age (per 1-year increase)	1.09	1.03–1.15		0.003	0.080	0.080	1.18	1.09–1.28		< 0.001	0.187	0.187
Positive APOE ε4 status	2.34	1.10–5.00		0.028	0.126	0.046	6.20	2371–16.2		< 0.001	0.329	0.142
Step 2: center (ADC = reference)				0.001	0.271	0.145				0.562	0.339	0.010
DELCODE	6.54	2.14–20.0		0.001			0.841	0.241–2.94		0.786		
IDIBAPS	5.78	2.14–15.6		0.001			1.53	0.476–4.89		0.477		
Model variables	Abnormal CSF-ptau181 (> 53 pg/ml)						Abnormal Aβ42 and abnormal ptau181					
Step 1: covariates												
Age (per 1-year increase)	Not selected						1.15	1.05–1.26		0.002	0.117	0.117
Positive APOE ε4 status	2.66	1.28–5.54		0.009	0.069	0.069	5.23	1.80–15.2		0.002	0.234	0.117
Step 2: center (ADC = reference)				0.728	0.075	0.006				0.393	0.255	0.021
DELCODE	0.983	0.401–2.41		0.970			1.68	0.374–7.54		0.499		
IDIBAPS	1352	0.574–3.19		0.490			2.60	0.643–10.5		0.180		

*R*^2^ and Δ*R*^2^ give an estimate of explained variance of the full model and each predictor’s contribution of explained variance within the model, respectively

No values are reported for nonsignificant predictor variables in step 1 of the models, as we used a forward selection process at this step

*Aβ* amyloid beta, *ADC* Amsterdam Dementia Cohort, *APOE* apolipoprotein E, *CI* confidence interval, *CSF* cerebrospinal fluid, *DELCODE* German Center for Neurodegenerative Diseases (DZNE), *IDIBAPS* l’Institut d’Investigacions Biomèdiques August Pi i Sunyer Hospital Clinic Barcelona, *OR* odds ratio, *ptau181* tau phosphorylated at position 181

After adjusting for age and APOE status in step 1, we observed a significant center effect, such that both IDIBAPS (odds ratio (OR) 5.78, 95% confidence interval (CI) 2.14–15.6) and DELCODE (OR 6.54, 95% CI 2.14–20.0) had similarly increased risk of abnormal Aβ42 values compared with ADC.

Higher age and positive APOE status were also associated with higher likelihood of having abnormal CSF-tau as well as higher likelihood of fulfilling the CSF-based preclinical AD definition. For CSF-ptau181 abnormality, only APOE4 was a significant covariate. Other covariate effects were not observed for these markers (for tau: sex, score test χ^2^_(df = 1)_ = 0.08, *p* = 0.777; years of education, score test χ^2^_(df = 1)_ = 0.014, *p* = 0.906; evidence of subtle cognitive decline, score test χ^2^_(df = 1)_ = 0.254, *p* = 0.614; for ptau181: age, score test χ^2^_(df = 1)_ = 0.474, *p* = 0.491; sex, score test χ^2^_(df = 1)_ = 0.074, *p* = 0.785; years of education, score test χ^2^_(df = 1)_ = 0.033, *p* = 0.857; evidence of subtle cognitive decline, score test χ^2^_(df = 1)_ = 2.45, *p* = 0.118; for the CSF-based preclinical AD definition: sex, score test χ^2^_(df = 1)_ = 0.08, *p* = 0.777; years of education, score test χ^2^_(df = 1)_ = 0.014, *p* = 0.906; evidence of subtle cognitive decline, score test χ^2^_(df = 1)_ = 0.254, *p* = 0.614).

There was also no center effect for these markers (see Table 2).

The supplementary analysis with IADL and depressive symptomatology as additional predictors showed similar results with regard to the effects reported above. Subtle IADL deficits and depressive symptomatology were not associated with likelihood of abnormality in any biomarker.

## Discussion

The aim of the present study was to compare the prevalence of CSF biomarker abnormality across three different memory clinic samples that collaborate within the Euro-SCD project and to identify predictors of abnormal CSF from a set of variables available in all subsamples, such as age, APOE genotype, depressive symptomatology, and neuropsychological and IADL performance. In addition, we determined, by testing for center effects, whether there was significant between-center heterogeneity with regard to CSF abnormality after adjusting for the aforementioned factors.

Unadjusted for any covariates, we observed significantly different frequencies of biomarker abnormality between the samples only for CSF-Aβ42. DELCODE had the highest prevalence of Aβ42 abnormality (64%), followed by IDIBAPS (57%) and ADC (22%). Results of the logistic regression analyses suggest that this apparent heterogeneity in Aβ42 abnormality between the samples was to some extent explained by age and APOE4 status, two factors for which an association with likelihood of amyloid pathology is already well established (e.g., [29]). Age had a stronger influence on Aβ42 abnormality than APOE4 status (see Table 2 for each predictor’s contribution of explained variance in *R*^2^) and the samples differed more strongly in mean age compared with frequency of APOE4 status (see Table 1). With regard to the aim of achieving homogeneous and enriched rates of amyloid positivity across multiple SCD cohorts, our results suggest that these two factors need to be addressed in a harmonized case-definition protocol. This could mean definition of similar age strata or a minimum age cutoff (e.g., > 60 years) as inclusion criterion while positive APOE status could be used to define subgroups of SCD patients even further enriched for preclinical AD. On the other hand, APOE status can be used as a balancing factor when selecting samples across multiple sites since selection of SCD samples with equal rates of APOE4 will increase cross-sample homogeneity with regard to the presence of preclinical AD.

However, even after adjusting for age and APOE status, we did observe significant variance in the likelihood of amyloid positivity between the centers which was of equal magnitude other than the portion of variance explained by age and APOE (see Table 2). The presence of this center-effect after accounting for age and APOE suggests that there are further, unmeasured factors which differed between centers and influenced the likelihood of amyloid pathology. This may include different referral procedures to the memory clinics across the three countries and differences in the center-specific SCD recruitment protocols, for example the application of different criteria to exclude MCI. Recruitment methods and sources of referral pose a major challenge to standardize (e.g., due to country-specific differences in the healthcare system) and also to quantify in a way that their influence can be assessed and controlled for in future studies.

Moreover, quantitative and qualitative information of the exact nature of the SCD experience may give additional, valuable information with regard to (the differences in) AD risk, as already suggested by the “SCD plus” criteria [5, 30]. This could inform recruitment protocols for SCD case definition in research studies. In the present study, we could not analyze this due to lack of data since a common continuous measurement of SCD was not available and joint analysis of differing scales would require advanced psychometric (i.e., item-response theory) techniques for which the sample size of the present study was not sufficient. The association of specific questionnaire items with biomarkers of AD in SCD is investigated in an ongoing multicohort project of the SCD Initiative (“item analysis project” [5]). Furthermore, we will address the relationship of the “SCD plus” features with AD biomarkers in each of the Euro-SCD cohorts in a future joint study. For this study, subjects will be prospectively recruited at each site with a harmonized SCD case definition protocol that also includes common assessment of the SCD plus features.

It is further noteworthy that, besides the observed differences in Aβ42 abnormality, the relative frequency of those with abnormal ptau181 within the group of amyloid positives was relatively equal across samples as can be seen by comparing the frequency of Aβ42 abnormality with the frequency of combined Aβ42 and ptau181 abnormality in Table 1 (DELCODE, 64.3/21.4 = 2.85; IDIBAPS, 56.8/20.5 = 2.77; and ADC, 22.0/8.0 = 2.75). Thus, around every third SCD patient with amyloid abnormality also had abnormal ptau181 and would thus fulfill preclinical AD criteria according to the most recent guidelines [3]. At the same time, however, the total number of subjects with ptau181 abnormality in the ADC cohort is relatively high when seen in relation to its overall lower amyloid positivity. This may suggest that this sample also contained, to a higher degree, SCD patients with tau pathology in the absence of a prototypical, amyloid-induced, AD pathological process (“non-AD pathological change” [3]). However, this is speculative, and potential reasons remain elusive to us at the moment. In this regard it is also worth mentioning that a relatively higher frequency of ptau181 abnormality compared with Aβ42 abnormality has also been reported in an earlier longitudinal SCD study of the ADC cohort [31]. In this study, however, isolated increased tau/ptau181 was not associated with future cognitive decline, so it might be partly nonspecific.

Of note, the high prevalence of Aβ42 abnormality reported here for the SCD patients from DELCODE and IDIBAPS is higher compared with that reported for SCD patients in earlier reports using these cohorts [8, 9]. However, this can be explained by the lower (i.e., stricter) cutoffs applied in these aforementioned studies. Of note, the cutoff for the present analysis is based on reference data from the ADC cohort, Gaussian mixture modeling [16], and adjusted for cohort-nonspecific upward drift in Innotest results [17]. Thus, it was the most suited cutoff for our central CSF analysis and, although leading to the aforementioned discrepancies compared with earlier reports for DELCODE and IDIBAPS, does not introduce a bias for the between-center analysis in the present study. Furthermore, it has recently been shown that differences in storage time (DELCODE recruitment started more recently than ADC and IDIBAPS) has no clear effect on CSF Aβ42, tau, and ptau181 values [32]. Nevertheless, we still acknowledge that differences in CSF handling and storage or the shipment of DELCODE/IDIBAPS probes to the central CSF analysis in the ADC center may have influenced our results. However, taken together, we propose that our central CSF analysis is a clear strength of the present study, that is it improved the validity of our outcome data rather than posing a serious source of bias.

Subtle IADL deficits did not predict CSF biomarker abnormality in the present sample. Of note, SCD and MCI patients display (by definition) largely preserved IADL functions. Therefore, the fact that we did not observe an association of subtle IADL deficits with abnormal CSF markers in the present study might be due to limitations in measurement, as the FAQ and DAD scale are not designed to capture very subtle deficits. This is underlined by the finding of ceiling effects (i.e., “fully unimpaired”) in both scales. In fact, a recent study, using a more refined IADL measure, demonstrated an increase in IADL impairment across the spectrum from cognitively normal (without subjective cognitive complaints) to MCI, with SCD subject’s IADL performance lying in between [33]. This further highlights the importance of a refined characterization of very early functional deficits in this group.

Previous studies have also shown, albeit modest, associations between subtle cognitive performance deficits and AD biomarkers in cognitively normal samples not recruited through memory clinics (e.g., [34, 35]). While in the present, memory clinic-based study the average neuropsychological performance in each subsample was well above the range of MCI-level impairment, we observed differences regarding the frequency of subjects classified as having evidence of subtle cognitive decline according to the method of Edmonds and colleagues (see Methods section and [23]). While DELCODE (14%) and IDIBAPS (9%) had a similar rate of subjects with evidence of subtle cognitive decline, it was considerably higher in ADC with about 40% of subjects fulfilling the criteria. Of note, this pattern might reflect that operationalization of MCI as an exclusion criterion in IDIBAPS and DELCODE was very similar (i.e., based on predefined impairment cutoffs). This approach differs from that of ADC where definition of MCI was based on clinical judgment of the complete neuropsychological information rather than applying a specific algorithm or impairment cutoff. While both are valid and commonly used approaches to exclude MCI in the definition of SCD [5], they may still lead to heterogeneity across the resulting samples in terms of neuropsychological performance. However, despite this discrepancy, evidence of subtle cognitive decline was not associated with higher likelihood of CSF abnormality. These results are in line with data from a large cohort study that showed equal risk for incident AD dementia in individuals with unimpaired memory performance but memory concerns compared with those with a study diagnosis of “early MCI” (i.e., conceptually similar to the subtle cognitive decline operationalization used here) [30]. This challenges the usefulness of subtle cognitive deficits as a predictor for AD risk in pre-MCI samples. However, a possible alternative explanation for the negative finding, and likewise for the observed differences regarding evidence of subtle cognitive decline, might be that our operationalization relied on center-specific normative data (and, to some extent, on nonidentical tests per domain in each center). The resulting *z*-scores thus only represent relative deficit scores compared with each center’s specific normative data. As a consequence, their comparability in an absolute manner is not feasible. Limited comparability of different normative samples and the fact that norms might be differently accurate for different age strata preclude this. These factors may have stirred the between-center differences regarding the number of SCD patients meeting the subtle cognitive decline criteria and, likewise, may have precluded the finding of an association between subtle cognitive performance deficits and biomarker abnormality in the present sample.

With regard to homogenous SCD sample selection, close attention should therefore be paid to enabling a homogenous neuropsychological characterization (ideally including comparable norms) to enable a valid, neuropsychological MCI definition as an exclusion criterion across different samples. Application of such a common MCI criterion may further reduce heterogeneity with regard to the prevalence of preclinical AD. However, this needs to be empirically tested in a future multisite study.

## Conclusions

In summary, the results of the present study emphasize the need for harmonized SCD case-definition protocols for future studies on intervention and prevention in this promising target group. Age range, genetic risk factors, and cognitive functional status are important factors to be considered in the development of such protocols aiming to achieve similar enrichment rates of preclinical AD.

## Abbreviations

AD: Alzheimer’s disease
ADC: Amsterdam Dementia Cohort
APOE: Apolipoprotein E
Aβ: Amyloid beta
BNT: Boston Naming Test
CERAD: Consortium to establish a registry of Alzheimer’s disease
CI: Confidence interval
CSF: Cerebrospinal fluid
DAD: Disability Assessment for Dementia
DELCODE: German Center for Neurodegenerative Diseases multicenter Longitudinal Cognitive Impairment and Dementia Study
Euro-SCD: European initiative on harmonization of subjective cognitive decline in preclinical Alzheimer’s disease
FAQ: Functional Activities Questionnaire
FCSRT: Free and Cued Selective Reminding Test
GDS: Geriatric Depression Scale
HADS: Hospital Anxiety and Depression Scale
IADL: Instrumental activities of daily living
IDIBAPS: L’Institut d’Investigacions Biomèdiques August Pi i Sunyer Barcelona
M@T: Memory Alteration Test
MCI: Mild cognitive impairment
MMSE: Mini-Mental State Examination
MRI: Magnetic resonance imaging
NIA-AA: National Institute on Aging and the Alzheimer’s Association
OR: Odds ratio
ptau181: Tau phosphorylated at position 181
RVLT: Rey Verbal Learning Test
SCD: Subjective cognitive decline
SD: Standard deviation
TMT: Trail-Making Test
t-tau: Total tau
VuMC: VU University Medical Center in Amsterdam
